# NEO412: A temozolomide analog with transdermal activity in melanoma *in vitro* and *in vivo*

**DOI:** 10.18632/oncotarget.26443

**Published:** 2018-12-11

**Authors:** Steve Swenson, Catalina Silva-Hirschberg, Weijun Wang, Anupam Singh, Florence M. Hofman, Kristen L. Chen, Axel H. Schönthal, Thomas C. Chen

**Affiliations:** ^1^ Department of Neurosurgery, Keck School of Medicine, University of Southern California, Los Angeles, CA, USA; ^2^ Department of Molecular Microbiology & Immunology, Keck School of Medicine, University of Southern California, Los Angeles, CA, USA; ^3^ Department of Pathology, Keck School of Medicine, University of Southern California, Los Angeles, CA, USA; ^4^ Medical College of Wisconsin, Milwaukee, WI, USA; ^5^ Department of Orthopedic Surgery, Keck School of Medicine, University of Southern California, Los Angeles, CA, USA

**Keywords:** perillyl alcohol, temozolomide, transdermal, melanoma *in situ*, linoleic acid

## Abstract

Despite new treatments introduced over the past several years, metastatic melanoma remains difficult to cure. Although melanoma *in situ* (MIS) has better prognosis, it relies heavily on thorough surgical excision, where ill-defined margins can pose a challenge to successful removal, potentially leading to invasive melanoma. As well, MIS in the head and neck area can create serious aesthetic concerns with regard to the surgical defect and substantial scar formation. Toward improved treatment of localized melanoma, including the targeting of unrecognized invasive components, we have been studying a novel agent, NEO412, designed for transdermal application. NEO412 is a tripartite agent that was created by covalent conjugation of three bioactive agents: temozolomide (TMZ, an alkylating agent), perillyl alcohol (POH, a naturally occurring monoterpene with anticancer properties), and linoleic acid (LA, an omega-6 essential fatty acid). We investigated the anti-melanoma potency of NEO412 *in vitro* and in mouse models *in vivo*. The *in vitro* results showed that NEO412 effectively killed melanoma cells, including TMZ-resistant and BRAF mutant ones, through DNA alkylation and subsequent apoptosis. *in vivo*, NEO412 inhibited tumor growth when applied topically to the skin of tumor-bearing animals, and this effect involved a combination of increased tumor cell death with decreased blood vessel development. At the same time, drug-treated mice continued to thrive, and there was no apparent damage to normal skin in response to daily drug applications. Combined, our results present NEO412 as a potentially promising new treatment for cutaneous melanoma, in particular MIS, deserving of further study.

## INTRODUCTION

Melanoma is the most dangerous type of skin cancer. It is estimated that there will be over 91,270 newly diagnosed cases in 2018 and an estimated 9,320 deaths [1]. Treatment of melanoma generally involves surgical removal of the tumors, and in advanced cases, biopsy of lymph nodes is performed as well [2]. With standard surgical excision the recurrence rate approaches 50% due to narrow surgical margins routinely used [3]. Systemic chemotherapy and immunotherapy are some of the standard treatment options in late stage disease, but the toxic side effects and recurrence secondary to chemo- and immunoresistance hinder its clinical usefulness [4]. Approximately 50% of all melanomas harbor BRAF mutations, and a number of agents are in development or have recently been approved to treat melanoma by targeting mutant BRAF [5]. In addition to targeting BRAF, several immunotherapies are in, or moving toward, the clinic focusing on immune checkpoint inhibitor antibodies [6]. A select few topical chemotherapeutics are being investigated, but whether there is better local tumor control with topical chemotherapy after surgical excision, thereby decreasing systemic metastasis, is not fully known [7]. While some of the older chemotherapeutic treatments for melanoma are considered outdated in light of the immunotherapies and BRAF checkpoint inhibitors, the body of evidence shows that the older therapies do have value [8].

One such chemotherapeutic is temozolomide, the current standard of chemotherapeutic care for malignant glioma [9]. It exerts its antitumor activity through the ability to create methyl adducts within DNA [10], most importantly O6-methylguanine (O6-meG), which triggers cell cycle-dependent DNA damage and cell death [11]. However, O6-methylguanine DNA-methyltransferase (MGMT, a DNA repair enzyme) can remove these methylations, and tumor cells overexpressing MGMT display pronounced resistance to TMZ [12].

Perillyl alcohol (POH), a naturally occurring monoterpene, has been shown to exert an effective anti-tumor effect in a variety of preclinical cancer models, including those of the breast, pancreas, and lung [13–15]. However, it did not enter clinical practice, primarily because dose-limiting intestinal toxicity became evident in clinical trials with oral POH [16]. Intriguingly, phase I/II clinical studies in Brazil demonstrated that simple intranasal inhalation of POH was effective against recurrent glioblastoma, in the absence of detectable toxic events [17], and a similar Phase I/IIa trial was recently started in the US.

Based on predictions obtained from *in silico* modeling, we have covalently conjugated POH to TMZ, thereby generating the novel molecule NEO212. In our preclinical studies, NEO212 revealed striking anticancer activity that was significantly greater than that of TMZ or POH, either alone or combined as a mixture. As well, NEO212 displayed its anticancer potency in MGMT-positive cells that were unresponsive to treatment with TMZ. Altogether, the activity of NEO212 was established in a variety of *in vitro* systems and in different mouse tumor models [18–22]. As detailed in the current study, we have now created a transdermal NEO212-derivative via conjugation to the fatty acid linoleic acid (LA).

A number of studies have shown that isomers of conjugated LA display anti-proliferative effects in different tumor types such as breast, colon and skin [23–25]. While the precise mechanism of action is unclear, it appears that LA plays a role in regulating cell cycle checkpoints [26]. It is postulated that the growth-arresting properties of LA are the consequence of its ability to activate tumor suppressor p53, which in turn leads to a loss of cyclins D1 and E, as well as an increase in cyclin-dependent kinase inhibitors p21 and p27 and continued activity of retinoblastoma protein, which prevents G1/S transition [27].

Transdermal delivery has advantages over both oral and intravenous drug administration. In topical administration, the first-pass metabolism of the liver on the agent is eliminated, thus reducing the early liver metabolism of the agent, and increasing the effective concentration both at the site of application as well as in the circulation. While intravenous administration also eliminates the first-pass liver metabolism, topical transdermal delivery has the further advantages of avoiding potentially painful needle sticks, risk of infection, and time spent in an outpatient infusion center. Drug delivery across the skin is an extremely attractive route, due to the possibility of targeting skin diseases (topical) and for achieving systemic effects (transdermal uptake). Transdermal systems are non-invasive and can be self-administered. They also improve patient compliance and the systems are generally inexpensive.

In the present study, we expanded upon the preclinical promise of NEO212, and conjugated this compound to LA, thus creating a triple-conjugate consisting of TMZ, POH and LA, which we called NEO412. We hypothesized that this new molecule would support transdermal application and therefore might be suitable for the treatment of cutaneous melanoma, in particular MIS. In the following, we present the characterization of NEO412 in preclinical models of melanoma.

## RESULTS

### NEO412 is more cytotoxic than TMZ *in vitro*

**Figure 1:**
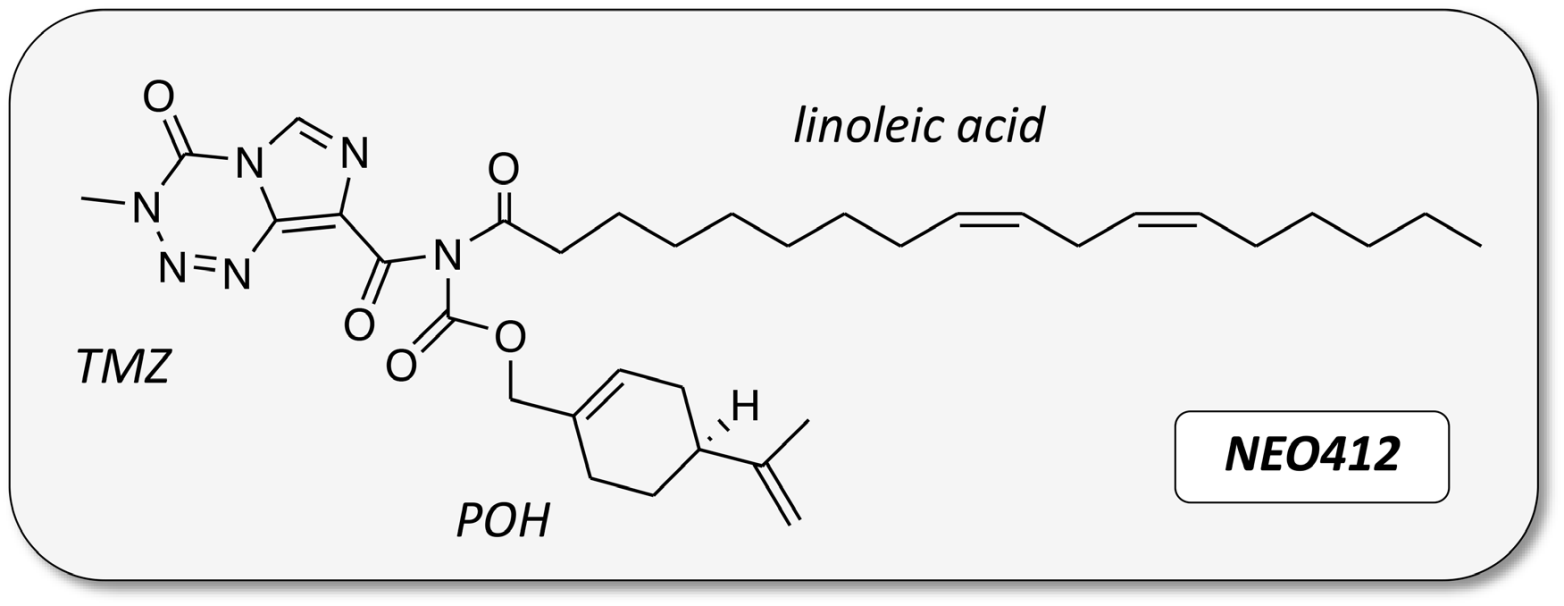
Chemical structure of NEO412 Temozolomide (TMZ) was covalently conjugated to perillyl alcohol (POH) and linoleic acid.

**Figure 2:**
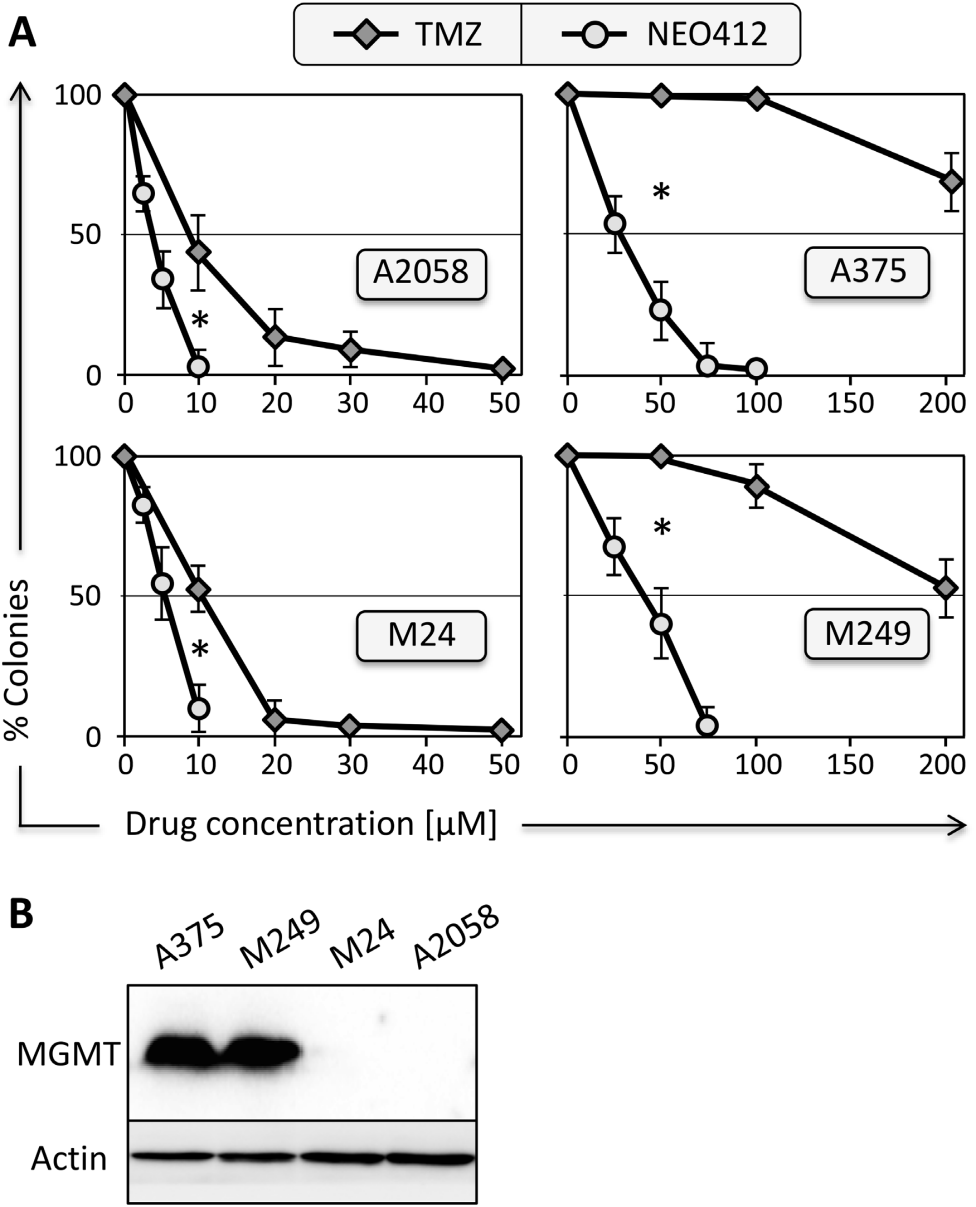
Survival of melanoma cell lines after drug treatment **(A)** Four different melanoma cell lines (as indicated) were exposed to increasing concentrations of NEO412 or TMZ for 48 hours, and long-term survival was determined by colony-formation assay two weeks later. In all graphs, colony formation by control cells (treated with vehicle only) was set at 100%; data points display mean (±SD; n≥3). Asterisks indicate *p*<0.01 between the respective same drug concentrations. **(B)** Cell lysates from the four cell lines were prepared and analyzed by Western blot with specific antibody to MGMT protein. Antibody to actin was used as a loading control.

An analog of TMZ was created by covalently linking both POH and linoleic acid to TMZ’s amide functionality. The chemical structure of this novel compound, called NEO412, is shown in Figure 1. The cytotoxic potency of NEO412 was analyzed in four different human melanoma cell lines (A2058, A375, M24, M249) and compared to the cytotoxicity of TMZ. We used colony-formation assays (CFAs), which determine the ability of cells to maintain their proliferative potency and spawn colonies of descendants. As shown in Figure 2A, NEO412 suppressed colony formation significantly more potently than TMZ in all four cell lines. Intriguingly, this differential was the greatest in cells expressing the DNA-repair protein MGMT (Figure 2B), which is known to confer resistance of tumor cells to treatment with TMZ. In the two MGMT-negative cell lines (A2058 and M24), the IC50 of NEO412 was about 5 μM, whereas the IC50 of TMZ was double this value. In the two MGMT-positive cell lines (A375 and M249), the IC50 of NEO412 increased to about 25 to 35 μM, but the IC50 of TMZ increased much more to >200 μM. Altogether, these results demonstrated greater *in vitro* antitumor activity of NEO412 as compared to TMZ, and the presence of MGMT was less protective against NEO412 than against TMZ.

**Figure 3:**
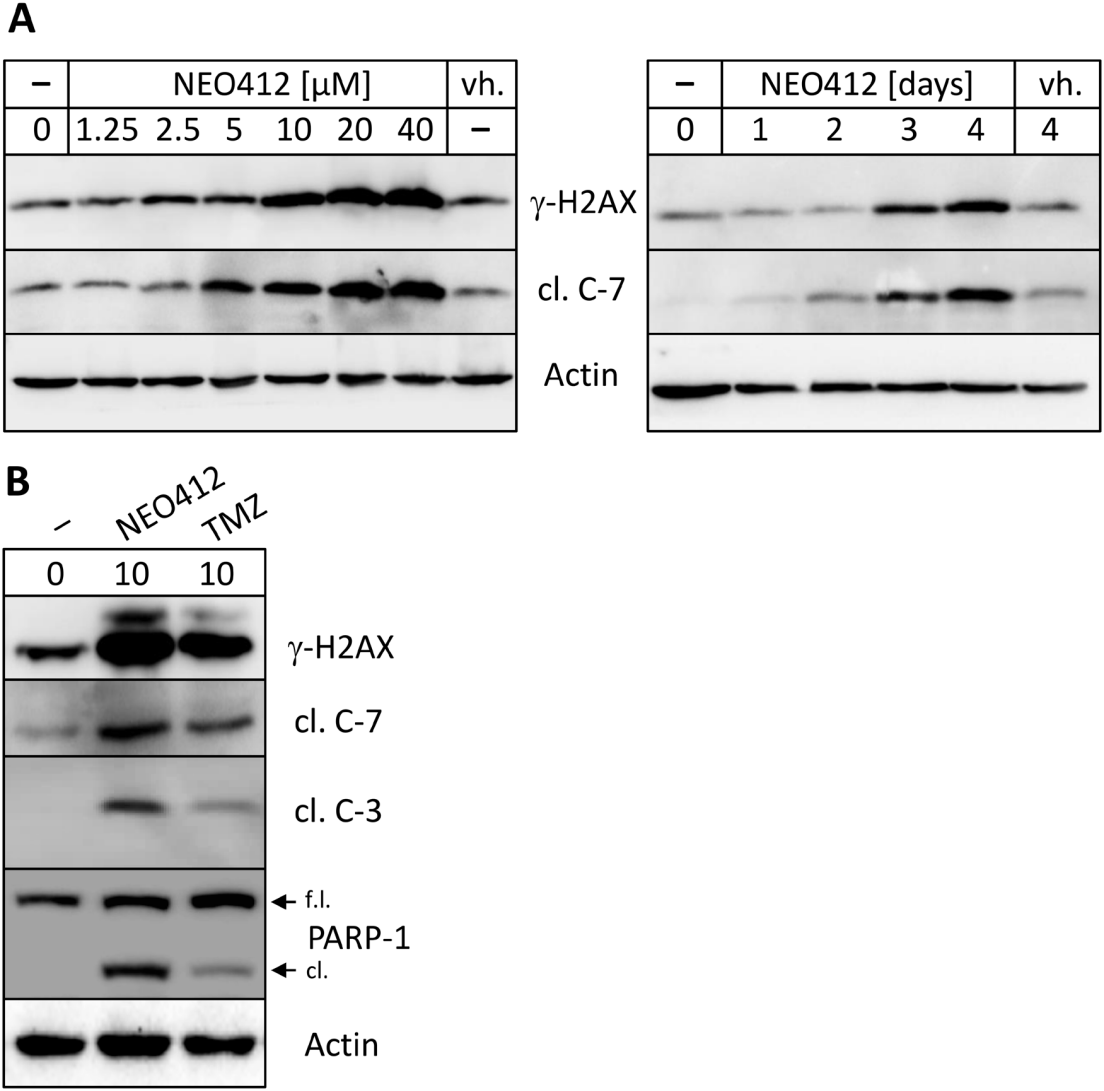
Drug effects on markers of DNA damage and apoptosis **(A)** A2058 cells were treated with increasing concentrations of NEO412 for 94 hours (left panel), or with 20 μM NEO412 for different time points (right panel). In parallel, cells were also treated with vehicle (vh.) alone. **(B)** A2058 cells were treated with 10 μM NEO412 or TMZ for 120 hours. In all instances, cells were harvested for Western blot analysis with antibodies against γ-H2AX (a marker for double-strand DNA damage), cleaved caspases 3 and 7 (cl. C-7; cl. C-7; markers for apoptosis), and cleaved (cl.) vs. full-length (f.l.) PARP (as an additional marker of apoptosis). Actin was used as a loading control.

In order to investigate the cytotoxic effects of NEO412 in greater detail, we characterized its impact on DNA damage and cell death at the molecular level. Lysates from drug-treated cells were subjected to Western blot analysis for γ-H2AX, a marker for DNA double-strand breaks, and for the emergence of cleaved (i.e. activated) caspase 7, a marker for apoptotic cell death. As shown in Figure 3A, treatment of A2058 melanoma cells with NEO412 resulted in increased levels of γ-H2AX protein, indicating accumulation of DNA strand breaks over the course of 3-4 days. Cleaved caspase 7 prominently emerged as well, in alignment with DNA damage. When used side-by-side at the same low concentration of 10 μM, NEO412 exerted greater impact on these markers than TMZ (Figure 3B). This was further validated by inclusion of two additional markers of apoptotic cell death, cleaved caspase 3 and cleavage of poly(ADP-ribose) polymerase 1 (PARP-1). Altogether, these markers indicated that the DNA-damaging and apoptosis-inducing ability of TMZ was preserved in NEO412, and that NEO412 was more potent than TMZ *in vitro*.

### Cytotoxic effect of NEO412 involves O6-guanine methylation

**Figure 4:**
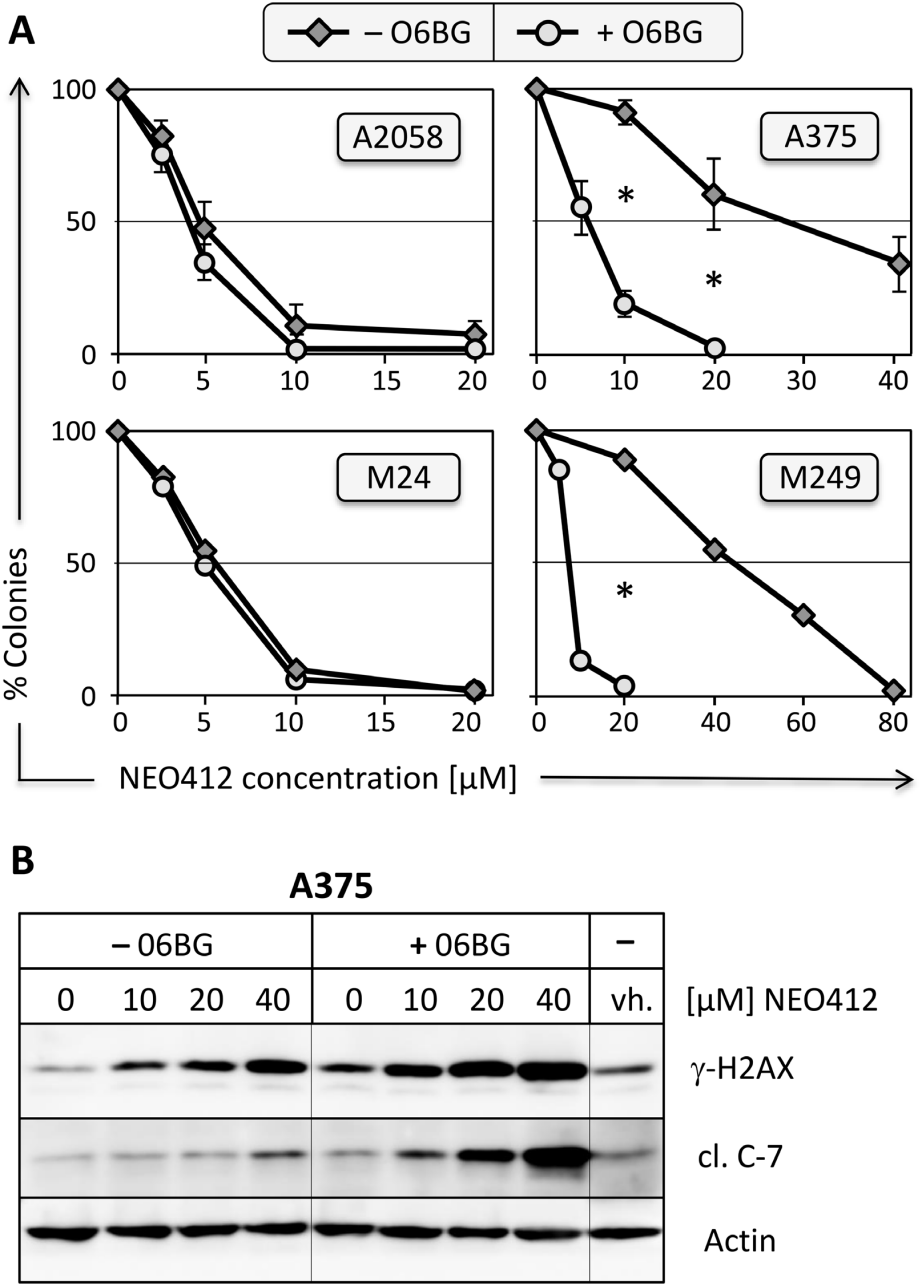
Effect of MGMT-inhibitor O6-benzylguanine **(A)** Two MGMT-negative (A2058 and M24) and two MGMT-positive (A375 and M249) melanoma cell lines were pre-incubated with or without 15 μM O6-BG for 30 minutes, followed by the addition of increasing concentrations of NEO412. Ten to twelve days later, colonies were stained and counted. In all graphs, colony formation by control cells (treated with vehicle or O6-BG only) was set at 100%; graphs with error bars display mean (±SD) from 3 independent experiments; graphs without error bars show the average from two independent experiments. Asterisks indicate *p*<0.01 between the respective same drug concentrations in A375 and M249 cells. For A2058 and M24 cells, differences were not statistically significant. **(B)** A375 cells were pre-incubated with or without 15 μM O6-BG for 30 minutes, followed by the addition of increasing concentrations of NEO412. Control cells received vehicle (vh.) only. Three days later, cellular lysates were prepared and analyzed by Western blot for DNA damage marker γ-H2AX and apoptosis marker cleaved caspase 7 (cl. C-7). Actin was used as a loading control in all blots.

MGMT is known to very effectively repair O6-methylguanine lesions set by TMZ, thereby providing profound resistance against cell killing by TMZ. As results shown in Figure 2 had suggested an influence of MGMT on the potency of NEO412, we investigated this correlation in greater detail. The four cell lines used above were treated with NEO412 in the presence or absence of O6-benzylguanine (O6-BG), a potent inhibitor of MGMT function, and cell survival was determined. As presented in Figure 4A, the inclusion of O6-BG had no impact on NEO412 toxicity in MGMT-negative cells (A2058 and M24), but greatly increased the sensitivity of MGMT-positive cells (A375 and M249). As well, when markers of DNA damage (γ-H2AX) and apoptosis (cleaved caspase 7) were investigated, we found that O6-BG increased the emergence of both of these markers when A375 cells were treated with NEO412 (Figure 4B). Altogether, these findings indicate that, similar to TMZ, methylation of O6-guanine represents a key cytotoxic target for NEO412. Although MGMT does provide some resistance against NEO412, this protective impact is smaller than the powerful protection of MGMT against TMZ.

### NEO412 is active *in vivo*

**Figure 5:**
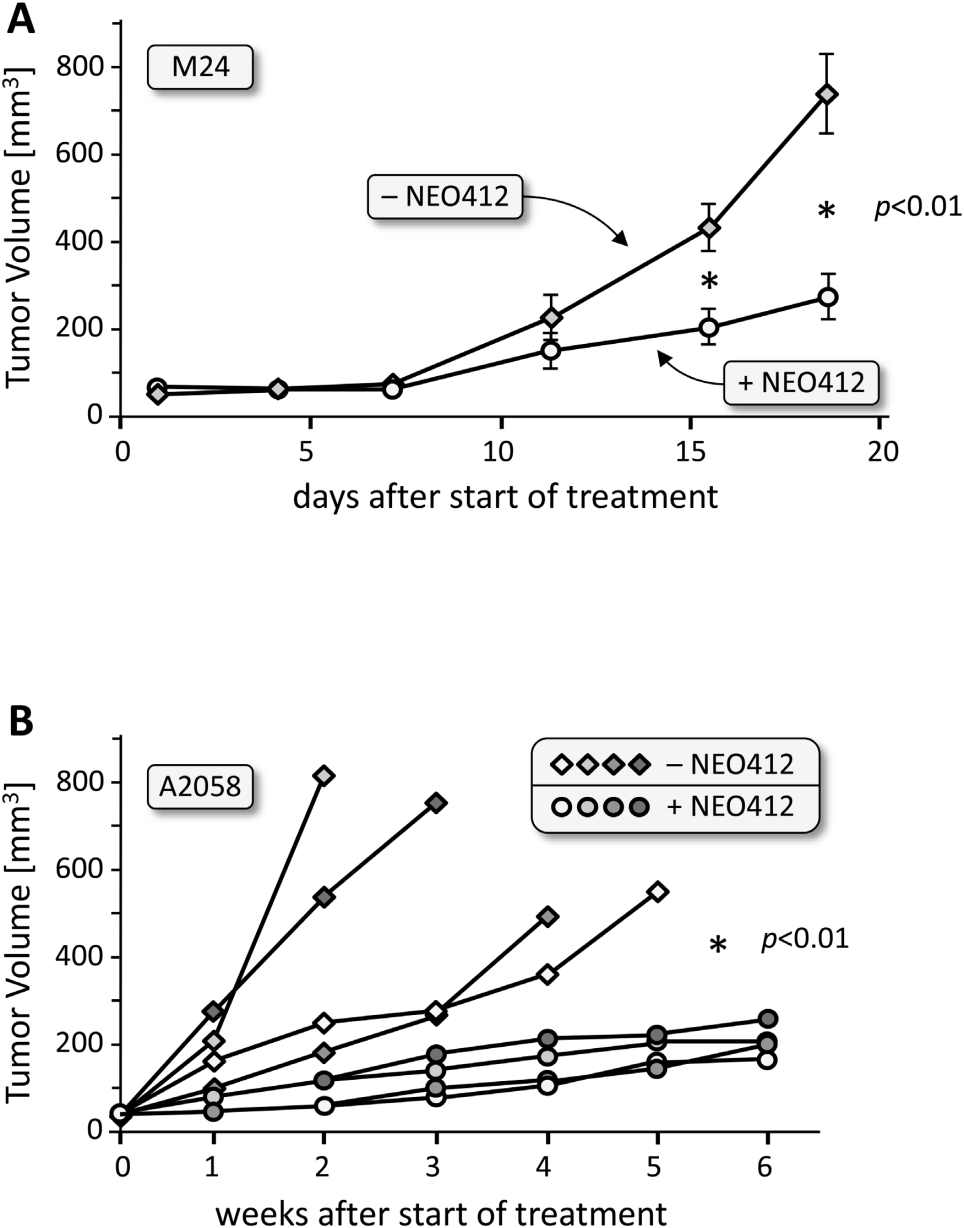
*In vivo* activity of transdermal NEO412 on MGMT-negative melanoma Melanoma cells were implanted subcutaneously, and after development of small nodules were treated with topical application of 25 mg/kg NEO412 or vehicle. **(A)** Results with M24 tumor cells (5 animals per group). **(B)** Results with A2058 tumor cells (4 animals per group). NEO412 exerted significant anti-tumor activity with no apparent skin toxicity. Due to rapid tumor growth (and cachexia) in the non-drug-treated animals, these mice had to be euthanized much earlier than those in the NEO412-treated group. In all cases, the difference between vehicle-treated and NEO412-treated groups was statistically significant (*p*<0.01).

**Figure 6:**
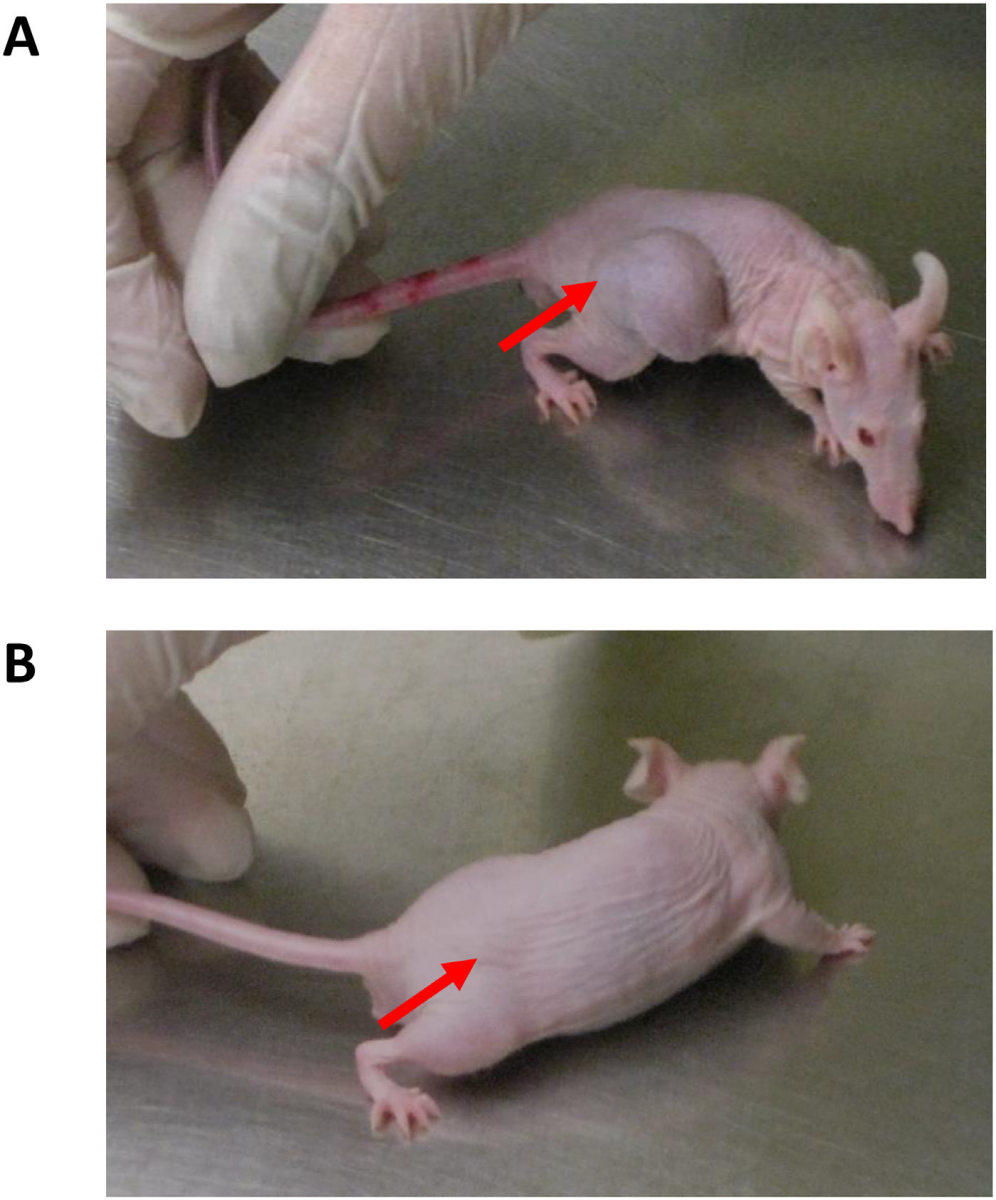
Visual differences of transdermal NEO412 on MGMT-negative melanoma Representative mice are shown and area of A2058 tumor cell implantation is indicated by red arrow. **(A)** Subcutaneous hip flank tumor displays a large mass and cachexia when treated with vehicle only. **(B)** NEO412-treated mouse displays significant tumor growth inhibition and maintains normal activity and body weight.

We next evaluated whether NEO412 would be able to exert its anticancer effects *in vivo*, utilizing transdermal delivery. Using two different MGMT-negative cell lines, M24 and A2058, animals harboring subcutaneous tumors were treated with a daily application of 25 mg/kg of NEO412 in a solution of 10% DMSO and 90% glycerol. NEO412 (or vehicle only) was placed on the skin above the tumor, spread with a spatula and allowed to dry. As presented in Figure 5A, animals with M24-derived tumors responded quite well to NEO412 treatment and presented with significantly slower tumor growth than animals treated with vehicle only. A similar result was obtained with mice carrying A2058-derived tumors, where NEO412 also very effectively inhibited tumor growth (Figure 5B). Figure 6 shows two representative mice to further illustrate the profound therapeutic impact of NEO412.

**Figure 7:**
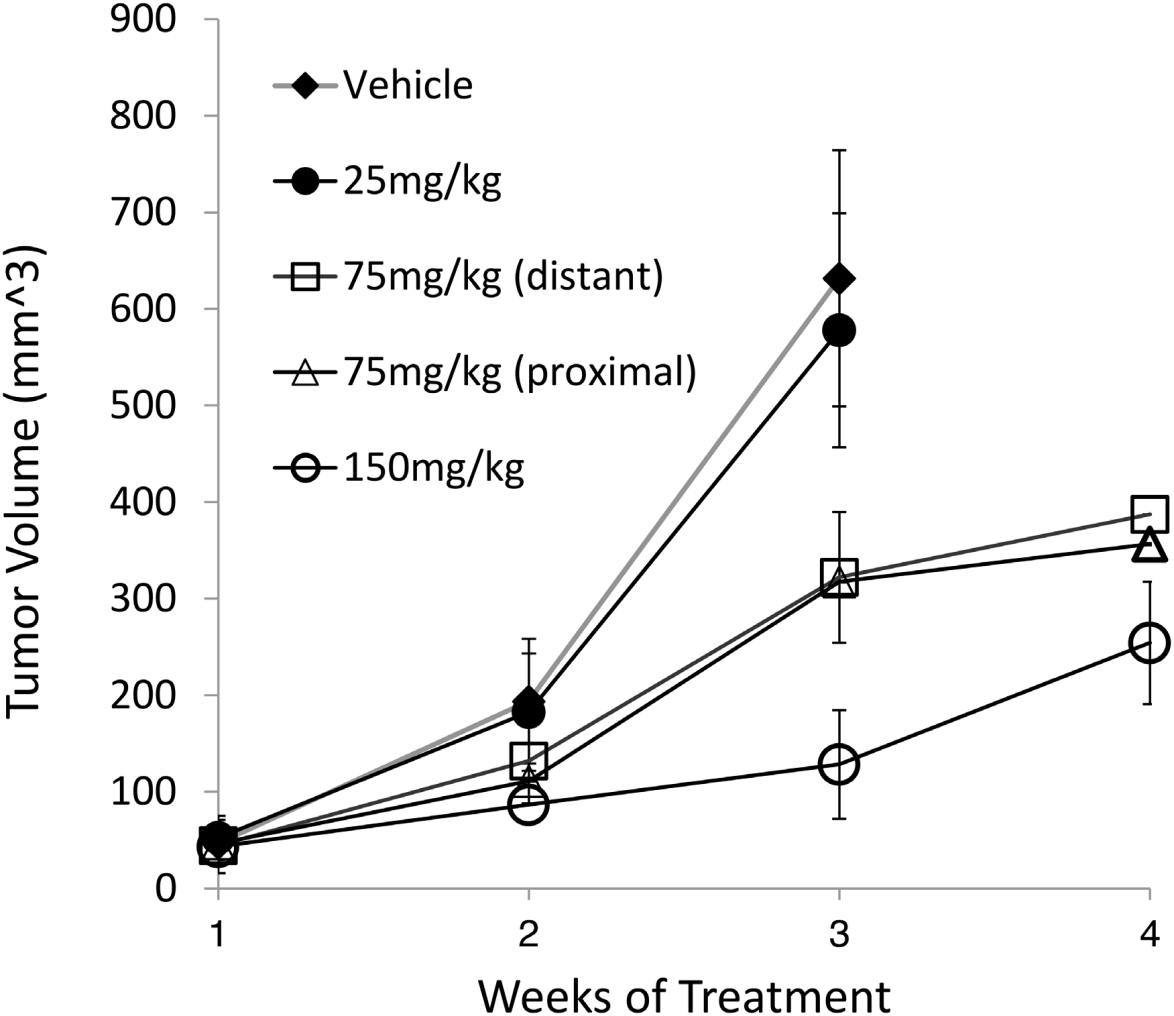
*In vivo* activity of transdermal NEO412 on MGMT-positive melanoma As an MGMT-positive model of melanoma, A375 cells were implanted in the hip flank of nude mice. Once the tumors were palpable, mice were separated into 5 groups of 5 animals each and treated once daily as follows: (i) vehicle only, (ii) 25 mg/kg NEO412, (iii) 75 mg/kg NEO412, and (iv) 150 mg/kg NEO412, where each treatment was applied to the skin directly above the tumor. In addition, (v) one group of mice received 75 mg/kg NEO412 that was applied to skin at a site distant to the tumor (neck area). All mice were weighed on Mondays and tumor size was measured on Thursday. Due to rapid tumor growth in the first two groups, these mice had to be euthanized after 3 weeks; therefore, no 4-week data points are available for them. At the 3-week time point, the difference in tumor size between the vehicle-treated group and the groups treated with 75 or 150 mg/kg NEO412 was statistically significant (*p*<0.01). There was no statistical difference between vehicle-treated animals and those receiving 25 mg/kg NEO412.

We then evaluated the efficacy of NEO412 in limiting the *in vivo* growth of A375, an MGMT-positive cell line. This model was carried out in the same fashion as above, except that different dosing levels of NEO412 were applied, namely 25, 75 and 150 mg/kg. In addition, we included a group in which 75 mg/kg NEO412 was applied to a site distant from the tumor, rather than to the skin directly above the tumor (proximal). The results are shown in Figure 7. NEO412 at 75 and 150 mg/kg displayed clear anti-tumor effects in this MGMT-positive tumor model. However, 25 mg/kg, which proved effective in the MGMT-negative models shown in Figure 5, did not unfold detectable therapeutic activity here. On the other hand, 75 mg/kg NEO412 was active, even when it was applied distal to the tumor site. Altogether, these results further established anti-melanoma activity of topical NEO412, although higher dosages seemed to be required in the case of MGMT-positive tumors; further, the activity of distally applied NEO412 pointed to a potential systemic component of this effect.

### Effects of topical NEO412 application on the skin

**Figure 8:**
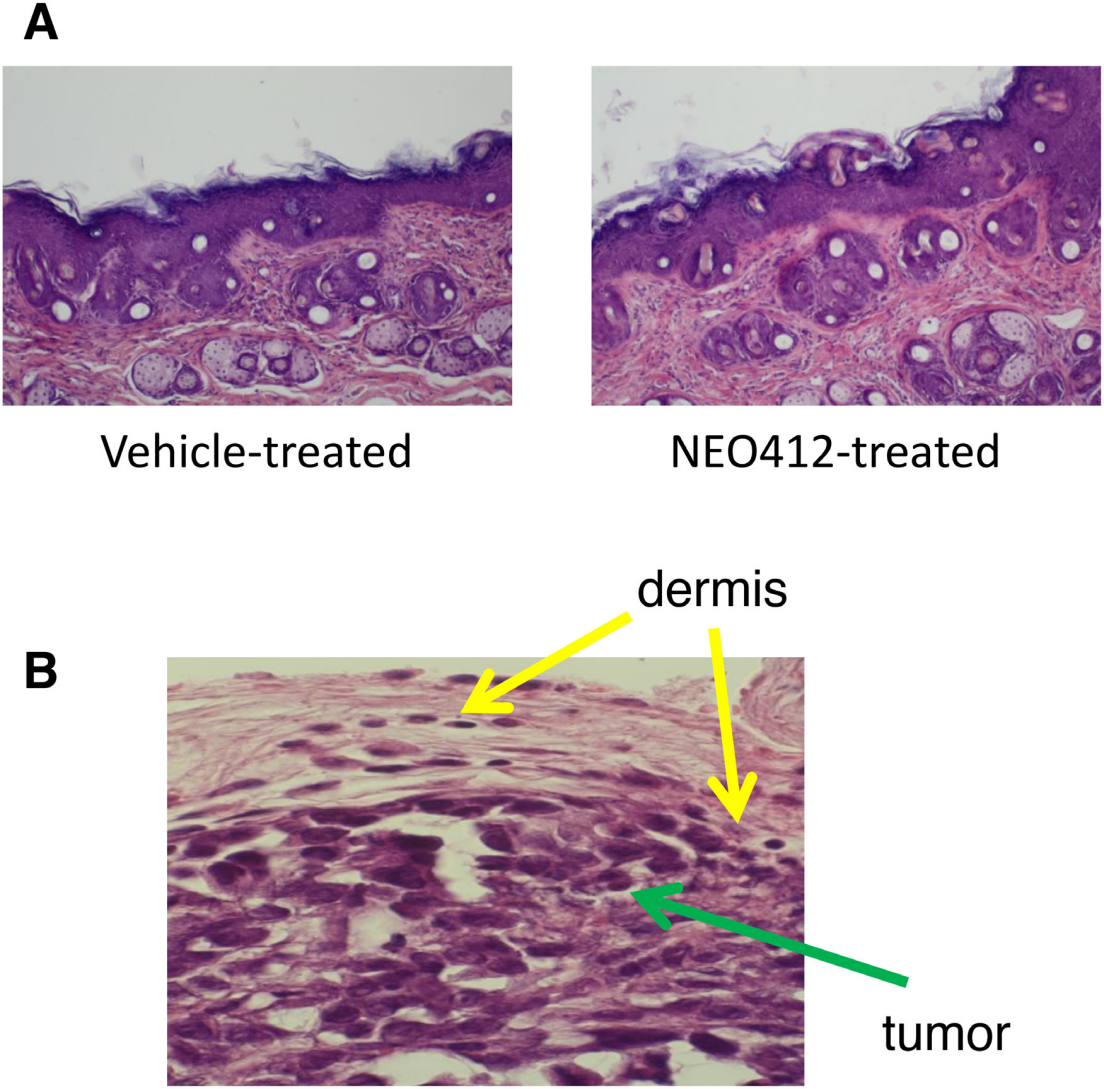
Microscopic evaluation of NEO412-treated skin as compared to vehicle-treated skin **(A)** Skin sections derived from mice that had received vehicle or NEO412 were stained with H&E and evaluated microscopically. No apparent differences between the two were observed (40x magnification; representative microphotographs are shown). **(B)** Local invasion of the tumor (green arrow) in the dermis (yellow arrow) is shown.

**Figure 9:**
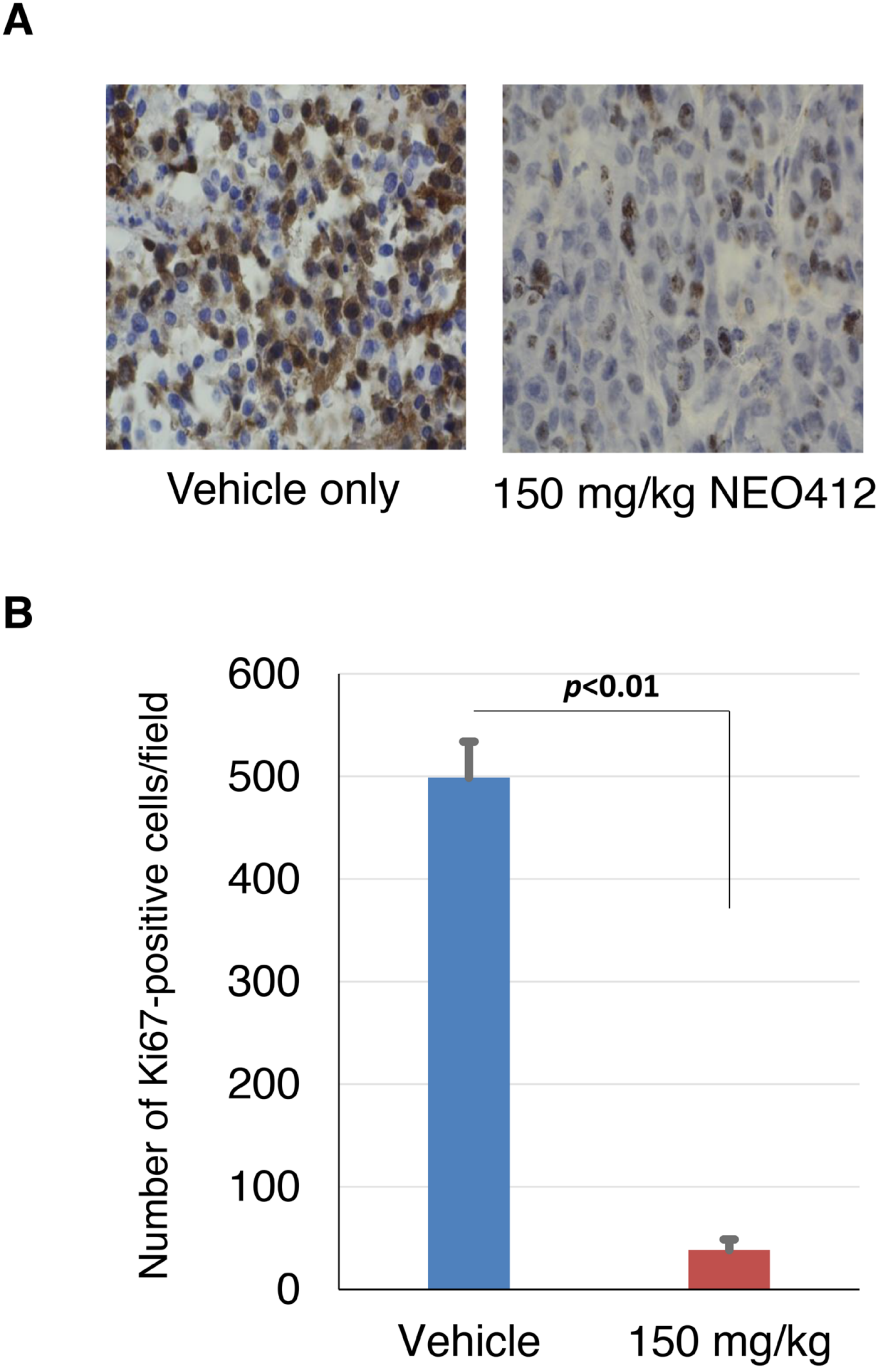
Ki-67 staining of treated tumors Tumor tissues from mice treated with vehicle only or treated with NEO412 were subjected to Ki-67 staining (as a marker for actively proliferating cells). **(A)** Shown are highly representative sections from 2 different animals. **(B)** The extent of Ki67 staining was quantitated by counting positive cells in 5 random microscopic fields each from 3 animals from both treatment groups. Shown is average (±SD) and statistically highly significant difference.

Topical application of NEO412 for periods of up to 8 weeks shows no noticeable effect on the skin both macroscopically and microscopically. Macroscopic observation is based on daily observation of the skin including lack of ulceration, little inflammation or redness and relatively no irritation. Microscopically, there is no observable effect of NEO412 on the skin when applied topically on a daily schedule (Figure 8A). The tumor is not restrained to the epidermis by spans both the epidermis and dermis making it more locally invasive (Figure 8B). Additionally, distant metastases were not observed on macroscopic examination at time of euthanasia (not shown). While there appeared to be no apparent adverse actions based on the topical application to skin, Ki-67 staining of the tumor sections, a marker for cell proliferation, revealed highly active proliferation in the tumor during vehicle treatment (Figure 9A). However, in response to NEO412, there was a >87% decrease in Ki-67-positive cells (Figure 9B), clearly indicating anticancer activity of this compound.

### NEO412 exerts anti-angiogenic activity

**Figure 10:**
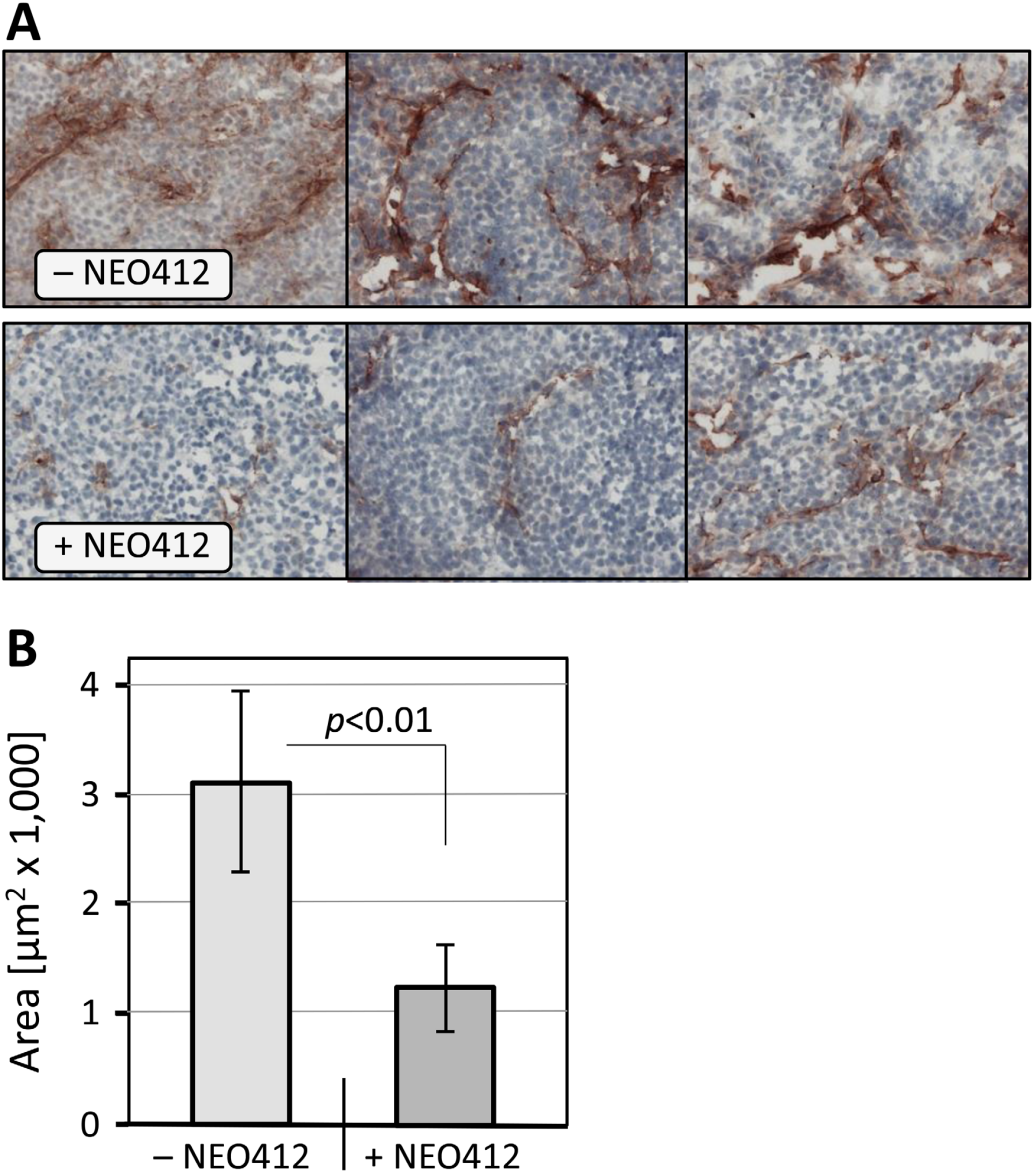
Microvessel density in melanoma tissue Tumor tissues were collected from mice described in legend to Figure 5. Immunohistochemistry with blood vessel marker anti-CD31/PECAM-1 was performed as detailed in Materials and Methods. **(A)** Representative photomicrographs of histological specimens from A2058 tumor tissues are shown. Note weaker CD31 staining in tissues from NEO412-treated animals. Similar differences were observed with specimens from M24 tumors. **(B)** Quantitative presentation of MVD from A2058 and M24 tumors combined. We compared 3 vehicle-treated mice to 3 drug-treated mice with A2058 tumors, and the same number of mice with M24 tumors, and used Image J to quantify the extent of CD31-positive staining. While differences within each cell line were significant (*p*<0.05), integrating both cell lines provided even higher significance (*p*<0.01).

To further characterize the *in vivo* anti-tumor activity of NEO412, we investigated its impact on blood vessel formation in tumor tissues from those animals described in Figure 6, where implanted A2058 or M24 melanoma cells had been subjected to transdermal treatment with NEO412 or vehicle only. Histological specimens were stained for CD-31 (PECAM-1, platelet endothelial cell adhesion molecule 1), a cell surface marker identifying endothelial cells. As shown in Figure 10A, the tumor treated with vehicle demonstrated more pronounced CD31 staining as compared to tumors from NEO412-treated mice. Quantitative analysis of these differences revealed a statistically significant (p<0.01) difference of nearly 3-fold between NEO412 and vehicle-treated tumors (Figure 10B). Thus, NEO412 effectively blocks vascular expansion in the tumor, thereby decreasing tumor progression.

## DISCUSSION

In this study, we demonstrated the anti-melanoma effect of NEO412 in cell culture, as well as by topical application to tumor-bearing mice *in vivo*. The cytotoxic effect of NEO412 unfolded even in cells with mutated BRAF (A375, A2058, M249) and in cells that were drug-resistant to TMZ treatment, based on their overexpression of the DNA repair enzyme MGMT (A375, M249). Based on these observations, NEO412 should be further studied as a candidate for topical melanoma therapy.

In this study, we have shown that NEO412, a conjugate derived from TMZ, linoleic acid and POH displays significant anti-tumor activity regardless of MGMT status, and displays no overtly observable side effects. We have demonstrated that NEO412 exhibited its cytotoxicity against melanoma cells *in vivo* when the agent is applied to the skin directly above the tumor as well as at distant sites and the agent is effective in a dose dependent manner in TMZ resistant cells (MGMT positive). The activity of NEO412 in both *in vitro* assays as well as the three different animal models make the agent a promising therapeutic agent against melanoma.

Topical application of drugs, notably to treat tumors of the skin has several distinct advantages from other routes of administration. One of these advantages is the ability to generate locally high concentrations of the drug at the site of the tumor. While oral dosing is available, this form of administration generates a systemic high concentration even if the drug accumulates at the site of the tumor. A high systemic concentration can exacerbate any potential side effects while local delivery reduces this possibility. As a caveat to these considerations, we also observed therapeutic impact of topical NEO412 when it was applied to skin distant from the tumor site. This outcome suggests that topical NEO412 also exerts some systemic effects, which in the context of melanoma could be advantageous. However, this aspect requires further study, in particular with regard to a potential concentration gradient between the proximal site of drug application and distant lesions.

We have not studied topical application of TMZ or NEO212 (TMZ conjugated to perillyl alcohol), but based on analysis of their chemical structures they were not deemed suitable for transdermal delivery. We hypothesized that a long-chain fatty acid would be required for efficient transdermal delivery, which was supported by prior reports, e.g., studies with docosahexaenoic acid [28]. Our results show that dermal application of NEO412 results in anticancer activity, indicating that our novel molecule indeed can be delivered through the skin.

The primary treatment of melanomas is surgical excision and prevention of metastatic disease. MIS in particular relies heavily on surgical intervention, although the optimal width of margins has remained somewhat controversial. While 5-mm margins represented a long-standing consensus, they appear to be inadequate, and newer studies have recommended the inclusion of up to 9 mm of normal-appearing skin, similar to that recommended for early invasive melanoma [29]. An additional topic for debate is the most appropriate type of surgical procedure, Moh’s micrographic surgery (MMS) vs. wide local excision (WLE). Compared to WLE, MMS represents a more elaborate approach that involves intraoperative frozen-section examination of the entire cutaneous margin for tumor cells. However, a very recent review of a database of 662 MIS patients treated with either WLE or MMS found no significant differences in the recurrence rate, overall survival, or melanoma-specific survival between the two groups [30].

Surgical intervention and optimal width of margins are critical decisions to prevent transformation of MIS into invasive melanoma, which has significant impact on morbidity and mortality [31]. Unfortunately, as more and more MIS is diagnosed in the head and neck region, there are instances where surgery may not be an option when the lesions are very large or less well defined. In such cases, negative margins may not be achievable without grossly disfiguring the patients. As well, some patients might have comorbidities that preclude a surgical option. Topical treatments could represent an alternative. Here, imiquimod cream was evaluated in several uncontrolled studies and case reports, with appealing results. However, due to the slow growth of MIS and relatively short-term follow up in these studies, it has been difficult to provide adequate proof of therapeutic activity of this treatment modality [32]. As discussed elsewhere [32], use of imiquimod might be risky as MIS can have a hidden invasive component that is not eliminated by this type of topical treatment; there are cases where MIS has progressed to invasive melanoma after treatment with imiquimod. In comparison, based on the observations in our animal models with the use of malignant melanoma cells, we would deem NEO412 effective also against any hidden invasive component, and thus more potent than currently available topicals. However, this prediction awaits further study and confirmation.

In our melanoma models, local invasion from the epidermis to the dermis is observed. This is to be expected due to the very thin epidermal layer in immunodeficient mice and the volume of cells injected to establish a primary tumor. While invasion to the local dermis is present, upon macroscopic examination no distant metastases are apparent. The treatment with NEO412 is able to penetrate the dermal layer as well as the surface and displays efficacy in limiting disease spread and progression on a local scale.

Our study provides proof of principle that NEO412 is effective against melanomas *in vitro* and *in vivo*, in both MGMT-positive and -negative tumors. As melanomas are the most aggressive skin cancer, we anticipate that NEO 412 will also be effective in other types of skin cancers, such as basal cell carcinoma and cutaneous T cell lymphomas. Those experiments are currently in progress.

Moreover, the concept of local adjunctive treatment in melanomas has been gaining increasing traction recently. Theurich et al. recently demonstrated that local adjunctive radiation therapy (i.e., external radiotherapy, electrochemotherapy, or internal radiotherapy) in conjunction with ipiluminab in melanoma patients can at least double their overall survival time [33]. Although we also believe that wide surgical excision with a margin is crucial for melanoma excision, there are still a number of patients for whom that has not been achieved. We are currently extending our studies to include a model in which there is residual melanoma after surgical excision. Our goal is to determine in future investigations whether NEO412 can be used to sterilize the surgical wound of any residual melanoma cells.

## MATERIALS AND METHODS

### Pharmacological agents

NEO412 was manufactured by Norac Pharma (Azusa, CA) and was kindly provided by NeOnc Technologies (Los Angeles, CA). It was diluted in DMSO to make a 100 mM stock solution. TMZ was obtained from the pharmacy at the University of Southern California (USC) and dissolved in DMSO to a concentration of 50 mM. POH, O6-BG and DMSO were purchased from Sigma-Aldrich (St. Louis, MO). In all cases of cell treatment, the final DMSO concentration in the culture medium never exceeded 1%, and was much lower in most cases. Stock solutions of all drugs were stored at –20°C.

### Cell lines

Human melanoma cell lines were propagated in DMEM supplemented with 10% fetal bovine serum (FBS), 100 U/mL penicillin, and 0.1 mg/mL streptomycin in a humidified incubator at 37°C and a 5% CO_2_ atmosphere. All cell culture reagents were provided by the Cell Culture Core Lab of the USC/Norris Comprehensive Cancer Center and prepared with raw materials from Cellgro/MediaTech (Manassas, VA). FBS was obtained from Omega Scientific (Tarzana, CA). Initial batches of cell lines were kindly provided by the laboratories of Alan Epstein (USC), Yves DeClerck (USC), and Ali Jazirehi (UCLA). Results with A2058 and A375 cells were confirmed with stocks obtained from the American Tissue Culture Collection (ATCC; Manassas, VA), and these cells were passaged for less than 6 months in our laboratory after receipt or resuscitation.

### Colony formation assay

Depending on the cell line (and plating efficiency), 300-600 cells were seeded into each well of a 6-well plate and treated as described in detail previously [34]. In the case of inclusion of O6-BG, cells were pre-treated with 15 μM O6-BG before the addition of NEO412. After 48 hours, drug-laced medium was removed and replaced with fresh medium containing 15 μM O6-BG. After 10-14 days, colonies (defined as groups of >50 cells) were visualized by staining for 4 hours with 1% methylene blue (in methanol), and then were counted.

### Immunoblots

Total cell lysates were analyzed by Western blot analysis as described earlier [35]. The primary antibodies were purchased from Cell Signaling Technology (Beverly, MA) or Santa Cruz Biotechnology, Inc. (Santa Cruz, CA) and used according to the manufacturers’ recommendations. All immunoblots were repeated at least once to confirm the results.

### Animal model

All animal protocols were approved by the Institutional Animal Care and Use Committee (IACUC) of USC, and all rules and regulations were followed during experimentation on animals. Athymic mice (Harlan, Inc., Indianapolis, IN) were implanted with 2x10^6^ cells into the right flank. About ten days later, once palpable tumors had developed, animals were assigned to different treatment groups. NEO412 was prepared daily from the stock solution at varying concentrations as a solution of 10% DMSO and 90% glycerol so that the applied volume in all cases was 50 μl. It was applied onto the skin either above the tumor or at a remote site distant from the tumor once daily. Application was performed by using a micropipette to draw the 50 μl, and the dot of liquid was placed on the skin and spread using the side of a sterile pipette tip. The animals were kept isolated in single cages following drug or vehicle application until dried to prevent other animals from licking the applied liquid. Once the topical had completely dried, the animals were returned to communal cages. The control group was treated in the same manner with vehicle only. Tumor volume was measured with calipers on a regular basis. In parallel, body weight was recorded.

### Tissue analysis

When animals were euthanized, skin and tumor tissues were collected and stored frozen or fixed in formalin. For the visualization of microvessels, frozen tumor sections were sectioned at 8 micron thickness, fixed in acetone, washed in phosphate-buffered saline, and blocked with Sea Block (Thermo Fisher Scientific, Waltham, MA). The sections were stained with anti-CD31 (PECAM-1/BD Pharmingen™; BD Biosciences, San Jose, CA), followed by incubation with secondary antibody, avidin-biotin peroxidase complex, and amino-ethylcarbazol substrate (VECTASTAIN ABC HRP kit; Vector Laboratories, Burlingame, CA) according to the manufacturer’s protocol. The samples were counterstained with hematoxylin for 1 minute. In parallel tissue sections, the same steps were performed in the absence of primary antibody as a control. MVD data were obtained by evaluating 10-20 random fields per section at 200x magnification from 3 different sections per mouse.

For histopathological skin analysis, samples were obtained from the areas both directly above the tumor (which were exposed to daily NEO412 or vehicle) as well as a distant site not treated with any topical. These samples were placed in 10% formalin for 24-48 hours. Thereafter, tissues were embedded in paraffin and cut into sections of 5 micron thickness. Slides were then stained using standard hematoxylin-eosin (H&E) stains, and evaluated under a light microscope. In addition, the sections were immunostained using standard techniques with Ki-67, a marker of cell proliferation, to evaluate the effect of NEO412 on cell proliferation within the tumor.

### Statistical analysis

All parametric data were analyzed using the Student *t*-test to calculate the significance values. A probability value (*p*) <0.05 was considered statistically significant.

## Abbreviations

CFA: colony formation assay
MGMT: O6-methylguanine DNA-methyltransferase
MVD: microvessel density
O6-BG: O6-benzylguanine
POH: perillyl alcohol
TMZ: temozolomide
